# Diameters and Fluorescence Calibration for Extracellular Vesicle Analyses by Flow Cytometry

**DOI:** 10.3390/ijms21217885

**Published:** 2020-10-23

**Authors:** Pasquale Simeone, Christian Celia, Giuseppina Bologna, Eva Ercolino, Laura Pierdomenico, Felisa Cilurzo, Rossella Grande, Francesca Diomede, Simone Vespa, Barbara Canonico, Michele Guescini, Vilberto Stocchi, Lavinia Vittoria Lotti, Maria Teresa Guagnano, Luisa Stellin, Stefano Papa, Oriana Trubiani, Marco Marchisio, Sebastiano Miscia, Paola Lanuti

**Affiliations:** 1 Department of Medicine and Aging Sciences, University “G. d’Annunzio”, Chieti-Pescara, 66100 Chieti, Italy; simeone.pasquale@gmail.com (P.S.); giuseppina.bologna@hotmail.it (G.B.); evin30@libero.it (E.E.); laura.pierdomenico@unich.it (L.P.); mariateresa.guagnano@unich.it (M.T.G.); luisa.stellin@unich.it (L.S.); s.miscia@unich.it (S.M.); p.lanuti@unich.it (P.L.); 2 Center for Advanced Studies and Technology (CAST), University “G. d’Annunzio”, Chieti-Pescara, 66100 Chieti, Italy; rossella.grande@unich.it (R.G.); sv85@libero.it (S.V.); 3 Department of Pharmacy, University “G. d’Annunzio”, Chieti-Pescara, 66100 Chieti, Italy; christian.celia@unich.it (C.C.); felisa.cilurzo@unich.it (F.C.); 4 Department of Medical, Oral and Biotechnological Sciences, University “G. d’Annunzio”, Chieti-Pescara, 66100 Chieti, Italy; francesca.diomede@unich.it (F.D.); oriana.trubiani@unich.it (O.T.); 5 Department of Biomolecular Sciences, University of Urbino Carlo Bo, 61029 Urbino, Italy; barbara.canonico@uniurb.it (B.C.); michele.guescini@uniurb.it (M.G.); vilberto.stocchi@uniurb.it (V.S.); stefano.papa@uniurb.it (S.P.); 6 Department of Experimental Medicine, “Sapienza”, University of Rome, 00161 Rome, Italy; laviniavittoria@uniroma1.it

**Keywords:** extracellular vesicles (EVs), flow cytometry, standardization, Rosetta bead system

## Abstract

Extracellular vesicles (EVs) play a crucial role in the intercellular crosstalk. Mesenchymal stem cell-derived EVs (MSC-EVs), displaying promising therapeutic roles, contribute to the strong rationale for developing EVs as an alternative therapeutic option. EV analysis still represents one of the major issues to be solved in order to translate the use of MSC-EV detection in clinical settings. Even if flow cytometry (FC) has been largely applied for EV studies, the lack of consensus on protocols for FC detection of EVs generated controversy. Standard FC procedures, based on scatter measurements, only allows the detection of the “tip of the iceberg” of all EVs. We applied an alternative FC approach based on the use of a trigger threshold on a fluorescence channel. The EV numbers obtained by the application of the fluorescence triggering resulted significantly higher in respect to them obtained from the same samples acquired by placing the threshold on the side scatter (SSC) channel. The analysis of EV concentrations carried out by three different standardized flow cytometers allowed us to achieve a high level of reproducibility (CV < 20%). By applying the here-reported method highly reproducible results in terms of EV analysis and concentration measurements were obtained.

## 1. Introduction

Under different stimuli, living cells produce and release many types of extracellular vesicles (EVs) into the extracellular milieu. The release of EVs is a well-documented and highly conserved process, described for prokaryotic and eukaryotic cells [1,2,3]. It has been demonstrated that EVs can be released by different pathogens [4] promoting their own dissemination [5,6]. In addition, mammalian EVs have been identified into many body fluids and have gained prominence as novel cell-to-cell communicators [7,8,9,10,11,12,13,14]. EVs have been also proposed as potential biomarkers for prognosis and diagnosis of different clinical conditions [12,13,15,16,17,18]. Recently, it has been observed that EVs represent a highly promising source of drug delivery vehicles [19]. In this context, a critical therapeutic role for EVs secreted by mesenchymal stem cells (MSCs) has been largely underlined by a recent body of literature [20]. It has been demonstrated that MSC-derived EVs display regenerative and anti-inflammatory properties, and have been demonstrated to be effective in a number of preclinical and clinical studies for the treatment of many diseases and injuries, such as graft-versus-host disease, acute cardiovascular events, autoimmune uveoretinitis, atopic dermatitis, neurodegenerative disorders, for treating cisplatin-induced nephrotoxicity, and autoimmune diseases [21,22,23,24,25,26,27]. The use of MSC-derived EVs, as an alternative to MSC themselves, produces many advantages, mainly related to their higher safety profiles. It has been observed that MSC-derived EVs have reduced levels of immunogenicity, they cross biological barriers, also avoiding complications linked to stem cell-induced ectopic tumor formation, entrapment in lung microvasculature, and immune rejection [13,20]. For these reasons, the rationale for developing EVs as an alternative therapeutic option results particularly strong.

Despite these evidences, the use of EVs and in particular of MSC-derived EVs in clinical settings will require the resolution of several critical issues, and, among them, one of the major challenges is related to the optimization of methods for rapid and accurate quantification and characterization of EVs.

In such a context, it must be underlined that multiple methods have been employed to detect EVs. Among techniques for single EV analysis, flow cytometry (FC) has a high potential for clinical application due to the high throughput and multiplex fluorescence capability [28]. However, the lack of consensus on EV detection and standardization protocols has resulted in controversy, and therefore, inconsistent FC results have been published [29,30,31]. By applying Mie theory to correct for refractive index differences between beads and EVs, it was shown that 24 out of 46 FCs in the field were unable to detect EVs smaller than 1,200 nm by triggering on scattering [30]. Moreover, alternative methods, such as atomic force microscopy (AFM) and nanoparticle tracking analysis (NTA), have shown that most EVs are smaller than 1,200 nm, confirming that by conventional FC only the “tip of the iceberg” can be measured [32,33]. Here we propose an optimized flow cytometry approach for EV identification and count on fresh samples of MSC-derived EVs, based on the use of Rosetta Calibration beads and the application of a fluorescent trigger threshold. Confirming recent data, we have demonstrated that triggering on a specific fluorescence channel, results in an improved analysis power and a higher EV count compared to triggering on scattered parameters [34,35,36,37]. Finally, a generic EV marker has been applied to the EV analysis carried out on three different flow cytometers, standardized as we have previously reported [38]. Results demonstrated that, by using the here-presented approach, a high level of reproducibility (CV < 20%) can be achieved [29,30,31].

We concluded that, by triggering on a fluorescent parameter, EV concentrations measured by flow cytometry become highly reproducible and accurate, therefore opening novel perspectives in assessing the clinical significance of EVs.

## 2. Results

### 2.1. Optimization of Scattered Parameters and EV Gating Strategy

**Figure 1:**
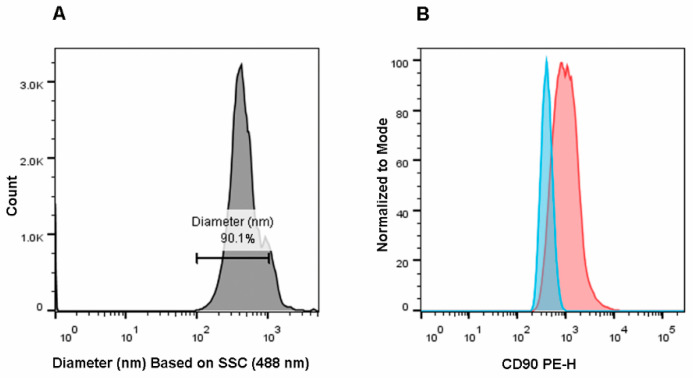
Mesenchymal stem cell (MSC)-derived extracellular vesicle (EV) analysis. (**A**) The size of events was obtained by transforming FSC files (.fcs) through the Rosetta Calibration system. The size of EVs was represented as a histogram and a gate covering EV dimensions was drawn (100–1000 nm). The percentage of EVs following in the EV diameter range is represented (90.1% of the acquired events). (**B**) The positivity to CD90 expression was verified: the histogram representing EVs stained by the anti-CD90 PE (Phycoerythrin, red) was overlaid to the histogram of the related isotype control (light blue).

Rosetta Calibration system adds the diameter of EVs in nm to “.fcs” files, thereby allowing to directly apply gates on EV size, instead of using scatter parameters. To select EVs, we gated the EV dimensions (“EVs 100–1000 nm”), as shown in the histogram of Figure 1A. Events displaying a diameter in the range 100–1000 nm were then analyzed for their CD90 expression. By using the corresponding isotype control, which is shown as a light blue histogram in Figure 1B, we found that the detected EV population expressed CD90 (red histogram), as demonstrated by the respective MFI (Mean Fluorescence Intensity) Ratio (>2.5) (Figure 1B).

As shown in Figure S1, pure CD90+ EVs (obtained by fluorescence-activated cell sorting) have average sizes comprised in the range of measurements between 100 nm–400 nm, thus demonstrating that the smallest EVs can be detected by FC and separated by cell sorting.

### 2.2. Trigger Threshold Optimization

**Figure 2:**
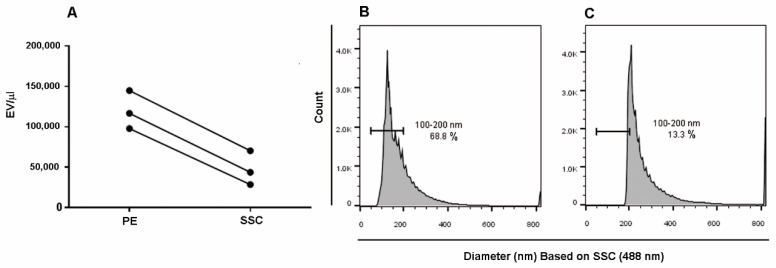
Side scatter (SSC)/phycoerythrin (PE) threshold comparison. (**A**) Three different MSC-derived EV supernatants were acquired using the same gating strategy by placing the threshold on PE or SSC. Counts obtained by the two triggering strategies were compared using the paired *t*-test (*p* = 0.0005; 2-tailed). (**B**,**C**) Dimensional gating encompassing a region of 100–200 nm was drawn on a diameter (nm) histogram (FCS files transformed by the Rosetta Calibration system) representing MSC-derived EVs acquired by thresholding on PE (**B**) or SSC (**C**).

We established the value for the trigger threshold on the PE (Phycoerythrin) channel which did not produce CD90+-EV loss, in order to measure the resolution sensitivity of the flow cytometer on the basis of PE-conjugated to the anti-CD90 which is a well-recognized MSC marker also expressed on MSC-derived EVs [39,40,41,42]. Three different samples of MSC-derived EVs (obtained from three different MSC clones) were then acquired triggering on side scatter (SSC) or on PE (CD90 detection channel). Figure 2A shows that fluorescence triggering results in significantly higher EV concentration (*p* = 0.0005, paired *t*-test) than triggering on SSC (*n* = 3; EV average concentration—PE threshold = 119,882 CD90+/μl vs. EV average concentration—SSC threshold = 47,582 CD90+/µL). By comparing diameter (nm) histograms obtained by assessing the PE threshold (Figure 2B) or the SSC threshold (Figure 2C), it became evident that the SSC threshold induced a significant loss of EVs (~50%). In detail, the smallest EVs (in the 100–200 nm area) resulted in being almost completely cut off by the SSC threshold.

### 2.3. Flow Cytometry Light Scattering Sensitivity Validation

**Figure 3:**
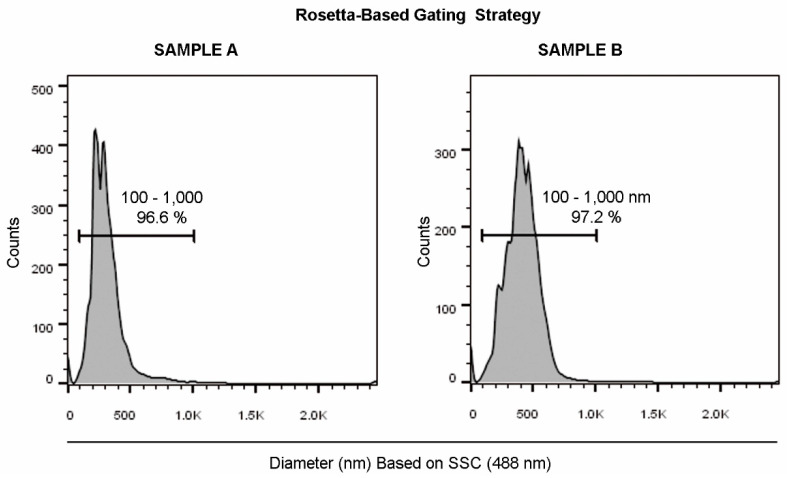
Rosetta bead system analysis of liposomes. The EV dimensional region (100–1000 nm) was defined on a diameter histogram (FCS files transformed by the Rosetta Calibration system). Sample A and B are represented.

To confirm the flow cytometry scattering sensitivity obtained by our approach, we have also synthesized Rhodamine-labeled liposomes of different diameters, as reported in the method section. Table 1, Figures S3 and S4 and Figure 3 show that liposomes from samples A and B displayed diameters in the range of small EVs, and DLS, NTA and Rosetta system analyses produced overlapping results.

### 2.4. Standardization of EV Analysis

The here described approach was standardized, by applying a fluorescent threshold on the PE emission channel, where PE was conjugated to the anti-CD90, a well-recognized MSC-derived EV marker. In this context, three instruments have been standardized (FACSVerse, FACSCanto II and FACSAria III; BD Biosciences, San Jose, CA, USA) as previously described [38]. As reported in the method section, also the threshold value was standardized and fine-tuned to detect almost no events when the filtered buffer, in the absence of the sample, was acquired. Notably, by setting the trigger threshold on the PE fluorescent channel, small particles and debris (that did not result stained) were not detected and not analyzed. The coefficient of variation value referred to the comparison of the EV concentration from the same MSC supernatant samples (*n* = 3), acquired by the three above mentioned standardized instruments, was <20.

### 2.5. EV Gating Strategy Based on a Lipophilic Cationic Dye

As an alternative approach, we also evaluated the possibility to stain EVs with a lipophilic cationic dye (LCD) recently identified as a generic EV tracer, highly useful when samples containing heterogeneous EV population need to be analyzed [43]. 

**Figure 4:**
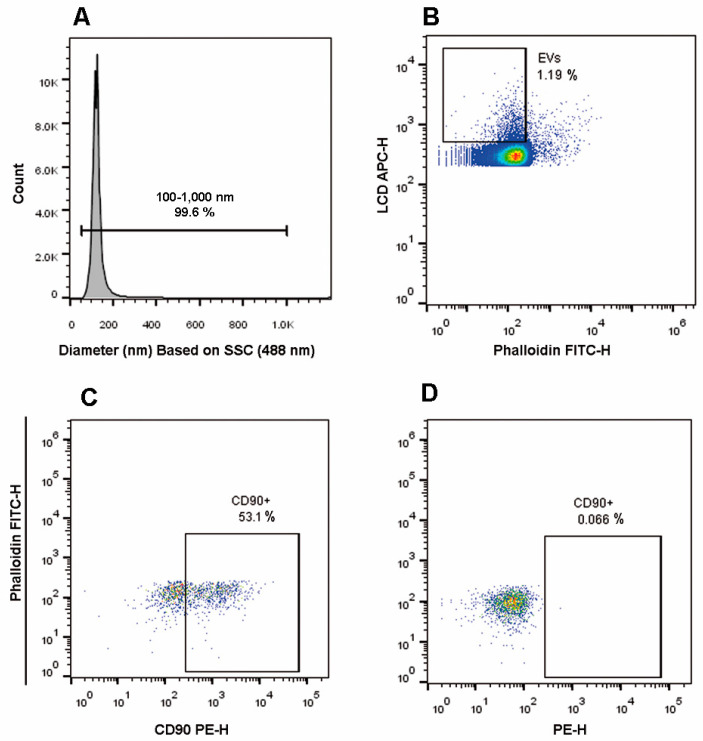
MSC-derived EV analysis. (**A**) The size of events was obtained by transforming .fcs files through the Rosetta Calibration system. The size of EVs was represented as a histogram and a gate covering EV dimensions was drawn (100–1000 nm). (**B**) Events displaying a diameter in the range 100–1000 nm were then analyzed on a Phalloidin FITC-H/LCD-H dot-plot and LCD+/Phalloidin- events were identified as EVs. (**C**) EVs were analyzed for their positivity to CD90 expression: (**D**) the dot-plot represents the FMO for CD90. The figure is representative of at least three separate experiments.

In this scenario, to select EVs, we set the trigger threshold on the channel in which the LCD emits (APC - Allophycocyanin), further gating the EV dimensions (“EVs 100–1000 nm”), as shown in the histogram of Figure 4A.

Events displaying a diameter in the range 100–1000 nm were then analyzed on a phalloidin FITC-H/LCD-H dot-plot (Figure 4B). Taking into account that LCD identifies the EV membranes and phalloidin stains damaged EVs, LCD+/Phalloidin- events were identified as intact EVs, which were further analyzed for their CD90 expression (Figure 4C), and CD90+ EVs were gated on the basis of the corresponding FMO (Fluorescence Minus One) control (Figure 4D) and the percentage of CD90+ EVs was anytime higher than 30%.

## 3. Discussion

EVs are cell-derived vesicles detectable in many biological fluids and involved in the intercellular crosstalk, as well as in several physio-pathological processes, i.e., coagulation, inflammation, angiogenesis, tumor invasion, immune responses and proteolysis [7,10,11,12,13,44,45]. For these reasons, EVs have gained a prominent role as potential biomarkers in different clinical conditions [8,12,13,46]. Furthermore, EVs have been also proposed as a promising source of drug delivery vehicles [13,19]. In particular, EVs released by mesenchymal stem cells (MSCs) have been demonstrated to have a critical regenerative and anti-inflammatory role, resulting effective in a number of preclinical and clinical studies for the treatment of many diseases and injuries [21,22,23,24,25,26,27]. 

Anyway, the identification of EVs remains a technical challenge due to their broad distribution and given that they are characterized by small diameters (100 nm–1 μm). In this context, flow cytometry (FC) represents the most promising technique for EV identification, count and sub-typing, because of its ability to quickly generate statistically robust results [28]. However, it has been demonstrated that 24 out of 46 FCs were unable to detect EVs smaller than 1,200 nm [30], while the lowest detection limit of current FCs is around 300 nm [47]. To overcome this limitation, setting up parameters in a specific manner, e.g., light scatter recovery in conventional instruments, has been proposed [47]. This is because the EV detection by FC was based on the calibration of scattered parameters with silica and/or polystyrene standard beads with specific average diameters. It has been also demonstrated that the refractive index of silica and polystyrene standard beads is higher than the refractive index of biological EVs. This gap might produce significant errors in the estimation of EV average diameters and numbers [44,48]. To obtain a more accurate strategy for EV detection, novel methods, based on the bead analyses and Mie scattering calculation algorithms, have been proposed [44,47]. EVs originate by budding from the respective parental cells, therefore, they expose the phenotype of the cell from which they stem. In this context, being CD90 an antigen highly expressed on the surface of the whole parental MSC population, it has been validated for the identification of MSC-derived EVs by the MISEV2018 position paper [39,40,41,42]. To reduce EV loss and preserve EV characteristics, EVs have been analyzed in untouched supernatants [16,49,50]. Here, the use of the Rosetta Calibration system was combined with the application of a trigger threshold on the channel in which the PE-conjugated anti-CD90 emits. In detail, we have demonstrated that EVs resulted in being distinguishable from the background noise. We have also demonstrated, confirming previously reported data, that, by triggering on fluorescence instead of placing the trigger threshold on scatter parameters, higher EV concentrations are measured, especially because of thresholding on SSC produces the loose of smallest EVs (100–200 nm) [34,35,36,37]. We have also shown that, by the approach here described, the gating based on the use of the Rosetta Calibration beads results highly accurate. This was demonstrated by the gating of fluorescent liposomes, that, being a polydisperse population of biological membranes, are ideal EV mimics. The combination of a standardized fluorescence triggering and the use of the Rosetta bead system on fresh supernatants allowed us to achieve highly reproducible results (CV < 20%). We have therefore demonstrated, for the first time, that using the Rosetta calibration system, in combination with a threshold placed on a specific fluorescent-parameter, allowed the identification of the MSC-derived EV compartment in fresh supernatant.

## 4. Materials and Methods

### 4.1. Obtainment of EVs from Mesenchymal Stem Cell (MSC) Cultures

Human mesenchymal stem cells were obtained, cultured and analyzed for their phenotypes as previously described [41,51,52]. This study (reference number: 266) was approved by the ethics committee for the Biomedical Research of Chieti and Pescara and of the University “G.d’Annunzio”, Chieti-Pescara (17 April 2017). In accordance with the Helsinki II Declaration, all involved subjects gave written informed consent before their inclusion in the study, and participants were identified by anonymized codes. Mesenchymal stem cell immunophenotypes and functional assays were carried out as previously reported, confirming their mesenchymal stem cell nature [39,41,51,52]. When used for the study of their supernatants, the percentage of dead cells (always less than 1%) was measured by flow cytometry, staining the cells with BD Via-Probe Cell Viability solution (Cat. 555815, BD Biosciences, San Jose, CA, USA).

### 4.2. Staining and Flow Cytometry Analysis of MSC-Derived EVs

We have analyzed the fresh supernatant of MSC cultures, containing EVs displaying the phenotype of their parental cells. In order to identify the best marker to study MSC-derived EVs, we tested different MSC specific antibodies, as listed in Table S1 and we confirmed that, given that CD90 represents a well-recognized pan-MSC- marker, it is also well expressed on MSC-derived EVs [42,51,53]. Therefore, for each sample, 100 μL of MSC supernatant were stained using 3 μL (0.006 μg/μL) of an anti-human CD90 conjugated to R-phycoerythrin (PE) (BD Biosciences, San Jose, CA, USA; Clone 5E10, Cat. 555596), or with the combination of CD90-PE (3 µL/test), a generic EV tracer (the Lipophilic Cationic Dye, LCD, 0.5 µL/test, BD Biosciences, San Jose, CA, USA, Custom kit Cat. 626267) and the FITC (Fluorescein isothiocyanate)-conjugated phalloidin (0.5 µL/test, BD Biosciences, San Jose, CA, USA, Custom kit Cat. 626267), which stains damaged EVs [43,54,55,56]. In any case, the anti-human CD90 that we used to identify MSC-derived EVs does not stain EVs eventually present in the bovine serum used for MSC cell cultures.

Samples were acquired after at least 30 min of staining (37 °C in the dark) by flow cytometry (FASCVerse, FACSCanto II, FACSAria III; all from BD Biosciences, San Jose, CA, USA).

As shown in Figure S2A, to avoid swarm effects, sample dilution (with filtered PBS) was established as recommended when a fluorescent threshold is used [35,36,37,57]. In any case, the flow rate resulted in event counts ≤ 2,000 events/second [58]. Diluted samples were then acquired, and 1 × 10^5^ events/sample were registered (FASCVerse, FACSCanto II, a FACSAria III fluorescence-activated cell sorter, BD Biosciences, San Jose, CA, USA). The trigger threshold was set at the PE channel (CD90 detection channel), or at the APC channel (LCD emission channel), while, to avoid EV loss, no threshold on scatter parameters was applied [16]. To evaluate the CD90 non-specific fluorescence, the relative isotype (Clone: MOPC-21, Cat. 555749, BD Biosciences, San Jose, CA, USA) or Fluorescence Minus One (FMO) controls were used [16,59,60,61]. Buffer-only, reagent-only and Triton X-100 1% control samples were acquired at the same settings used for analysis, measured using the same set time applied for the acquisition of stained samples [42].The unspecific background linked to the antibody aggregation and the immune complex formation was prevented by spinning the antibody stock solutions before their use (21,000 g, 12 min). The anti-CD90 PE-conjugated was titrated under the assay conditions (Figure S2B). 

Count differences between the acquisitions, obtained by placing the threshold on the PE channel or on the SSC (as recommended), were also evaluated.

Mean Fluorescence Intensity (MFI) ratios were calculated as previously described [38].

### 4.3. Flow Cytometry Assessment

To broadly setup scatter parameters for EV and liposome detection, Megamix-Plus beads (Biocytex, Marseille, France) were acquired according to manufacturer’s instructions (threshold on FITC channel). Height (H) signals and logarithmic or bi-exponential modes were selected for all parameters.

Instrument performances were monitored by the Cytometer Setup and Tracking Module and further validated by the acquisition of Rainbow Beads (BD Biosciences, San Jose, CA, USA ) [38,59,60]. Data were analyzed using FACSDiva v 6.1.3 (BD Biosciences, San Jose, CA, USA), FACSuite v 1.0.6.5230 (BD Biosciences, San Jose, CA, USA) and FlowJo v 10.0.7 (TreeStar, now Becton, Dickinson and Company, Ashland, OR, USA) software.

### 4.4. Rosetta Calibration for Diameter Assessment

Rosetta Calibration (Exometry, Amsterdam, The Netherlands) was used to establish the scatter to diameter relationship for the side scatter detector of the FACSVerse [30,62]. Briefly, Rosetta Calibration beads were run according to the manufacturer’s recommendations on the FACSVerse, using the same setting applied for EV detection but triggering on SSC. The relationship between liposomes or EVs and the respective diameter was automatically obtained by Mie theory, taking into account the optical configuration of the FACSVerse, and assuming a particle refractive index core of 1.40 for EVs and 1.34 for liposomes, respectively (Figure S2C) [30,62].

In detail, based on recent literature [30,62], we assumed that EVs have a 4 nm phospholipid membrane, with a lumen refractive index between water (1.34) and 1.40, and a shell refractive index between 1.46 (phospholipid) and 1.56 (phospholipid with proteins). Regarding liposomes, we know that they do not contain proteins, but they were charged by rhodamine, therefore a shell refractive index of 1.52 resulted in being appropriate for their characterization. For liposomes, we assumed a core refractive index of 1.34.

On the other hand, we also estimated the refractive indexes that we have used for EVs, by analyzing, with the Rosetta system, platelet samples as a biological control. Given that, by using 1.40 and 1.48 as core and shell refractive indexes, respectively, the Rosetta bead system exactly measured, for platelets, their expected diameters (~2,700 nm) [63].

### 4.5. Flow Cytometry Standardization

To explore the possibility to standardize EV counts, stained samples were also acquired by using three different flow cytometers (FACSVerse, FACSCanto II, and FACSAria III; San Jose, CA, USA). All three instruments were standardized on the PE channel using Sphero Rainbow Calibration Particles (BD Biosciences, San Jose, CA, USA, cat. 559123) as previously described [38] and Megamix-Plus beads (Biocytex, Marseille, France) to broadly set scatter parameters, according to the manufacturer’s recommendations. The threshold, applied on the PE channel, was regulated until just above the background counts while measuring buffer-only samples.

To define the obtained level of standardization, MSC-derived EVs from the same samples (*n* = 3) were counted by using Trucount tubes (BD Biosciences, San Jose, CA, USA), using as a stopping gate the bead number (200).

### 4.6. Fluorescence-Activated Cell Sorting of MSC-Derived EVs

EVs were stained as above reported, with LCD (0.5 µl/test, BD Biosciences, San Jose, CA, USA, Custom kit. Cat. 626267), phalloidin FITC-conjugated (0.5 µl/test, BD Biosciences, San Jose, CA, USA, Custom kit. Cat. 626267) and CD90-PE (3 µl/test). EVs were then separated (100 µm nozzle) from fresh supernatants based on their positivity pattern to CD90, by using a FACSAria III fluorescence-activated cell sorter (BD Biosciences, San Jose, CA, USA), equipped with three lasers (488, 633 and 375 nm, standard optical configuration). The post-sorting purity was assessed by using the same instrument (FACSAria III fluorescence-activated cell sorter, BD Biosciences, San Jose, CA, USA) and the same setting applied for MSC-derived EV separation, as recently recommended. Pure CD90+ EVs (97% of purity) were then analyzed by DLS as described here below.

### 4.7. Synthesis of Fluorescent Liposomes and DLS Analyses

Liposomes were synthesized by the thin-layer evaporation and small unilamellar vesicle extrusion by using Phospholipon^®^ 90G (PL90G, Lipoid LLC, NJ, USA): cholesterol (Chol, Merck Merck MilliporeSigma, St. Louis, MO, USA ) at the following molar ratio (7:3) [64]. Briefly, lipids were dissolved in chloroform/methanol (3:1 v/v) and the resulting lipid film, obtained by removing organic solvents by rotary evaporation (Heidolph Instruments, Schwabach, Germany), was hydrated using HEPES buffer (10 mM, pH = 7.4) [65]. Before hydration, the lipid film was vacuumed-dried overnight (12 h, 23 ± 1 °C); and lissamine rhodamine B 1,2-dihexadecanoyl-sn-glycero-3-phosphoethanolamine, triethylammonium salt (rhodamine DHPE, 100 μL corresponding to 0.1 molar ratio) (Molecular Probes, now Thermo Fisher Scientific, Waltham, MA, USA)) was added to the organic solution of lipids [64]. Multilamellar fluorescent liposomes were obtained with three heating/vortexing cycles (3 min each, 45 ± 0.5 °C, 700 rpm). Multilamellar fluorescent liposomes were then extruded through two stacked specific pore-size polycarbonate filters (Whatman Inc., NJ, USA) of 1000, 800, 600, 400, 200, 100 using a Lipex Biomembranes extruder (Northern Lipids Inc., Burnaby, BC, Canada). A 10-cycle extrusion (at 45 ± 0.5 °C) per filter size was performed to obtain a narrow size distribution of fluorescent liposomes [64].

Mean sizes and polydispersity index (PDI) and the Zeta-potential of fluorescent liposomes were physicochemically characterized using a Zetasizer Nano ZS (Malvern Instruments Ltd., Malvern, UK) as previously reported [66]. Parameters of the DLS analysis and laser Doppler electrophoresis were set-up according to the software settings as previously reported [66]. Multiple scattering was avoided by diluting samples in HEPES buffer (10 mM, pH = 7.4), prepared in Milli-Q water and filtered through 0.22 µm Millipore membranes. Disposable cuvettes for the size and the Z-potential (Malvern Instruments Ltd., Malvern, UK) measurements were used for 3 sets of 10 measurements of each sample and mean and standard deviation (S.D.) were calculated.

Liposomes features were also analyzed by Nanoparticle Tracking Analysis (NTA). For such a purpose, liposomes were diluted to approximately 1 mL of PBS, loaded into the sample chamber of an LM12 unit (Nanosight, Malvern, UK) and five videos of 60 s were recorded of each sample. The analysis was performed with NTA 3.1 software (Nanosight), and data are presented as the mean ± SE of the five video recordings. Control 100 and 200 nm beads were supplied by Malvern Instruments Ltd (Malvern, UK).

Liposomes obtained by using 100 nm (Sample A) and 200 nm (Sample B) extruder membranes were measured in the range from ~100 to ~300 nm, as demonstrated by the related DLS and NTA analyses (Table 1, Figure S3). This dimension range covers the critical region for EV detection by flow cytometry, therefore we used those liposome preparations in order to assess the resolution sensitivity of our flow cytometry measurements.

To evaluate liposome morphology, atomic force microscopy (AFM) and Transmission Electron Microscopy (TEM) analyses were carried out. For AFM evaluation, Liposomes were analyzed as already reported for EVs [67].

In order to obtain TEM analyses, negative staining protocols were carried out. In detail, 10 μL of suspended liposomal particles were placed on carbon-coated copper grids and adsorbed for about 5 min. Samples were then stained in a solution of 2% uranyl acetate for 10 seconds at room temperature. Excess of staining solution was removed by light absorption with filter papers. After air-drying the grids were examined with a digital Morgagni Philips transmission electron microscope (TEM), equipped with an image analysis software for morphometric analysis (FEI, Eindhoven, The Netherlands). TEM images showed liposome dimensions (for both samples A and B) overlapping with DLS and NTA results (Figure S4).

Liposomes were finally analyzed by flow cytometry. For each sample of liposomes, the stopping gate was set on “all events” and 1 × 10^4^ elements were acquired. Liposomes were registered at the same voltages used for Megamix-Plus and Rosetta Calibration beads. The trigger threshold was placed on the channel in which Rhodamine emits (PE-Channel).

### 4.8. Statistical Analysis

Statistical analyses were performed using GraphPad Prism 6 (GraphPad Software Inc., La Jolla, CA, USA) software. The comparison of EV concentrations was performed using a Paired *t*-test. Statistical significance was accepted at *p*  <  0.05 (two-tailed).

## 5. Conclusions

The FC ability to detect MSC-derived EVs resulted highly improved, given that, by avoiding any pre-analytical manipulation, as well as avoiding triggering on scattered parameters, no EV events are lost, thus making EV analyses by FC more reliable and reproducible. For our instruments, we have shown that MSC-derived EVs that are hidden under the “tip of the iceberg”, can be detected by selecting the optimal trigger threshold and detector. Unfortunately, not all the EV subtypes can be identified by recognized markers, but the use of promising generic EV tracers (such as LCD), combined to the here presented approach, improving the FC detection of EVs, may open new routes for the study of both eukaryotic and prokaryotic EVs in vitro, ex vivo, as well as in any clinical setting.

## Figures and Tables

**Table 1:** Liposomes characterization.

	NTA	DLS	Rosetta Beads
Sample A	Mean = 114.2 ± 1.3 nm Mode = 94.2 ± 3.2 nm D10: 75.0 ± 0.8 nm D50: 97.8 ± 0.6 nm D90: 150.6 ± 7.3 nm	PDI = 0.08 ± 0.01 Range = 100–110 nm Mean = 103.2 ± 2.4 nm Median = 103.2 ± 2.4 nm	Range=146–553 nm Mean = 317 nm
Sample B	Mean = 125.4 ± 2.7 nm Mode = 105.6 ± 6.4 nm D10: 87.6 ± 0.2 nm D50: 114.4 ± 1.1 nm D90: 156.8 ± 1.1 nm	PDI = 0.362 ± 0.034 Range = 158–270 nm Mean = 214 ± 7.2 nm Median = 214 ± 7.2 nm	Range=205–753 nm Mean = 442 nm
Nanoparticle Tracking Analysis (NTA); Dynamic Light Scattering (DLS).			

## Supplementary Materials

The following are available online at https://www.mdpi.com/1422-0067/21/21/7885/s1, Figure S1: Dynamic Light Scattering Analysis of MSC-derived EVs, Figure S2: Controls for EV analysis, Figure S3: DLS and NTA analyses of liposomes, Figure S4. Transmission electron microscopy analysis of Liposomes, Table S1: List of tested Reagents.

## Abbreviations

AFM	Atomic force microscopy
APC	Allophycocyanin
DLS	Dynamic Light Scattering
EVs	Extracellular vesicles
FC	Flow cytometry
FITC	Fluorescein isothiocyanate
FMO	Fluorescence Minus One
LCD	Lipophilic Cationic Dye
MFI	Mean Fluorescence Intensity
MSC	Mesenchymal stem cell
NTA	Nanoparticle Tracking Analysis
PDI	Polydispersity index
PE	Phycoerythrin
SSC	Side scatter
TEM	Transmission Electron Microscopy
