# Supplementary material for: Diameters and Fluorescence Calibration for Extracellular Vesicle Analyses by Flow Cytometry
Source: Int J Mol Sci. 2020 Oct 23;21(21):7885. doi: 10.3390/ijms21217885 (PMC7660682; doi:10.3390/ijms21217885)
Supplement: Supplementary file 1 [file ijms-21-07885-s001.pdf]

SUPPLEMENTARY MATERIALS

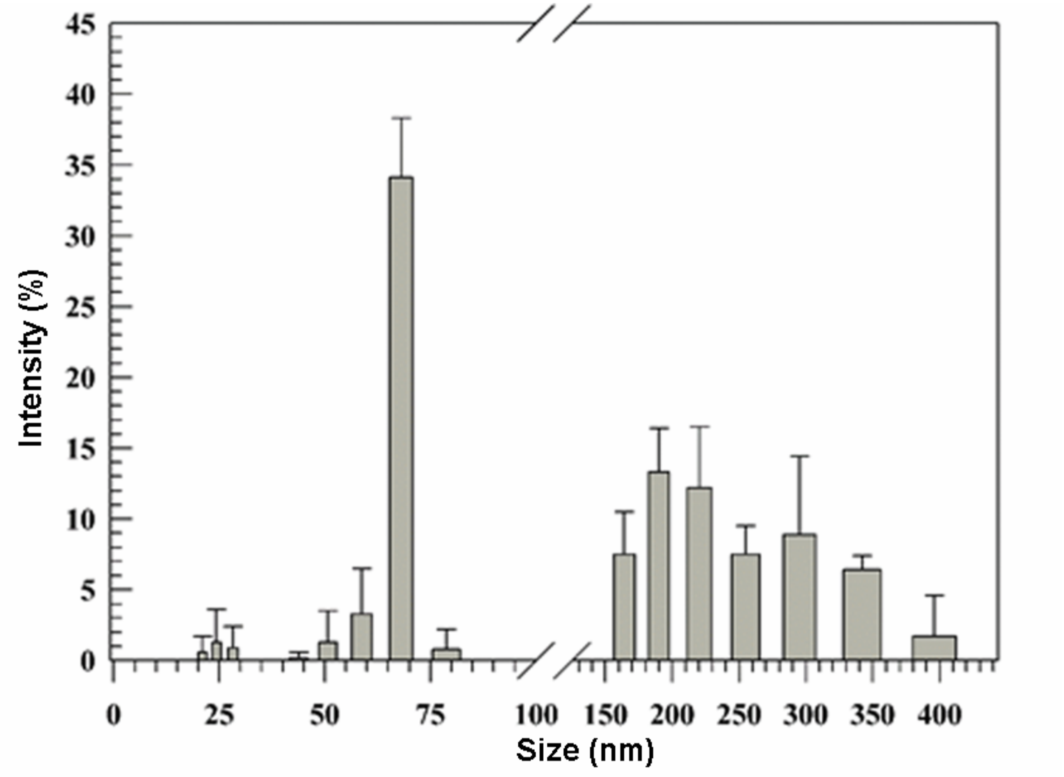

Supplementary Figure S1. Dynamic Light Scattering Analysis of MSC-derived EVs. The dynamic light scattering analysis of MSC-derived EVs, previously purified by the instrumental cell sorting, shows a heterogeneous bimodal distribution: smallest events (25 - 75 nm) representing the population of debris and biggest events (150-400 nm) that are the MSC-derived EV compartment. Figures are representative of five measurements as triplicates.

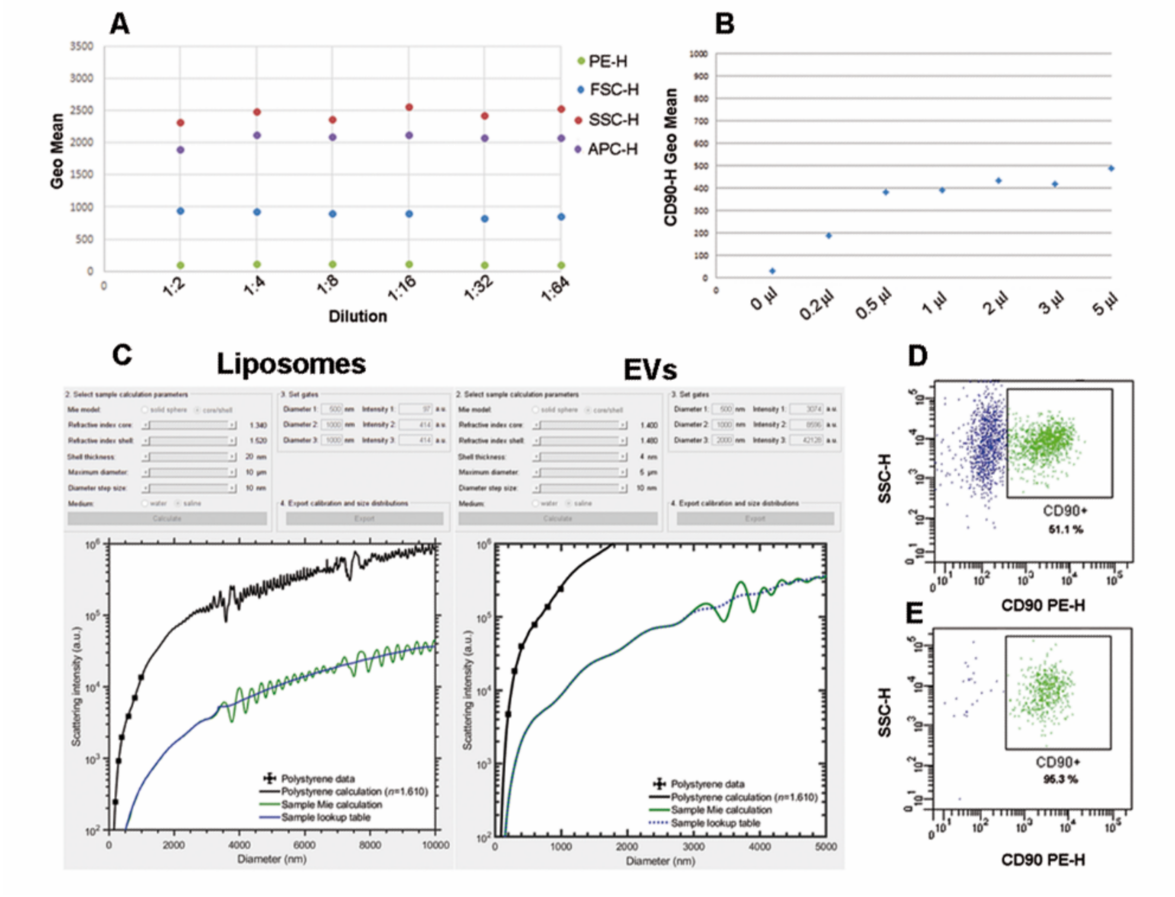

Supplementary Figure S2. Controls for EV analysis. A. Geo mean values for FSC-H (blue dots), SSC-H (red dots), CD90 PE-H (green dots) and LCD APC-H (purple dots) of LCD+/Phalloidin-/CD90+ MSC-derived EVs were reported for a number of serial dilutions. **B.** PE-conjugated anti-CD90 was titrated under assay conditions and the Geo Mean-H values were reported. **C.** Parameters for Mie model, refractive index core, refractive index shell, shell thickness, maximum diameter, diameter step diameter, and medium were reported for liposomes and EVs diameter calculation. Graphs show the relationship between the scattering intensity in arbitrary units (a.u.) and the diameter. The line shows a fit based on Mie theory for polystyrene beads and for EV/liposome samples taking into account the optical configuration of the instrument. **D.** EVs (LCD+/Phalloidin-/CD90+) were gated and separated by fluorescence-activated cell sorter (FACSAria III). **E**. The purity of MSC-derived EVs is shown (FACSAria III acquisition).

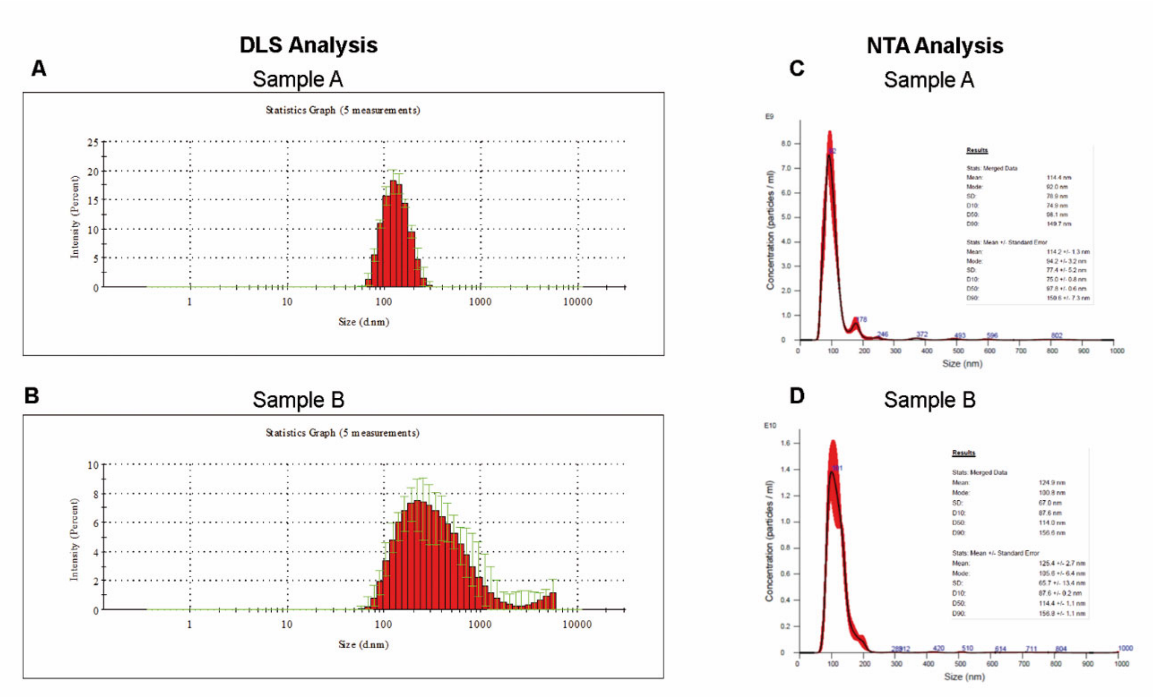

Supplementary Figure S3. DLS and NTA analyses of liposomes. Liposomes from Sample A (**A**) and from Sample B (**B**) were analysed by DLS analyses. Liposome dimensions from Sample A (**C**) and from Sample B (**D**) were analysed using a nanoparticle tracking assay, and results are represented as mean ± SEM.

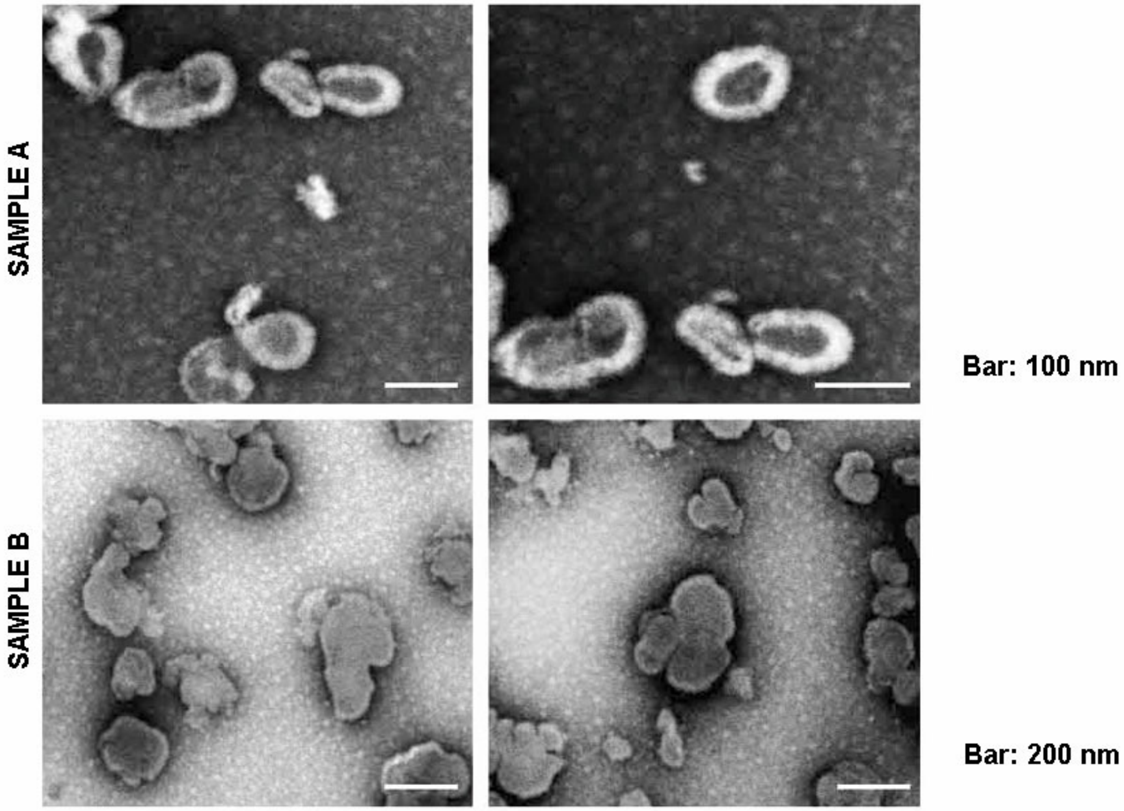

Supplementary Figure S4. Transmission electron microscopy analysis of Liposomes. Representative TEM images of spherical liposomes of different diameters detected by negative staining. Average diameters of liposomes were measured by an image analysis system. Scale bars: a) 100 nm; b) 200 nm.

**Supplementary Table S1. List of tested Reagents**			
Reagent	***Fluorochrome/Reagent***	***Vendor***	***Catalogue Number***
CD13	FITC	Ancell	162-040
CD29	PE-	BD Biosciences	555443
CD73	PE	BD Biosciences	550257
CD105	FITC	Ancell	326-040
FITC=Fluorescein isothiocyanate; PE= R-phycoerythrin.			
